# Lipoteichoic acid of *Streptococcus oralis* Uo5: a novel biochemical structure comprising an unusual phosphorylcholine substitution pattern compared to *Streptococcus pneumoniae*

**DOI:** 10.1038/srep16718

**Published:** 2015-11-18

**Authors:** Nicolas Gisch, Dominik Schwudke, Simone Thomsen, Nathalie Heß, Regine Hakenbeck, Dalia Denapaite

**Affiliations:** 1 Division of Bioanalytical Chemistry, Priority Area Infections, Research Center Borstel, Leibniz-Center for Medicine and Biosciences, Parkallee 1-40, 23845 Borstel, Germany; 2 Department of Microbiology, University of Kaiserslautern, Paul-Ehrlich Straße 24, 67663 Kaiserslautern, Germany

## Abstract

Members of the Mitis group of streptococci possess teichoic acids (TAs) as integral components of their cell wall that are unique among Gram-positive bacteria. Both, lipoteichoic (LTA) and wall teichoic acid, are formed by the same biosynthetic pathway, are of high complexity and contain phosphorylcholine (*P*-Cho) residues. These residues serve as anchors for choline-binding proteins (CBPs), some of which have been identified as virulence factors of the human pathogen *Streptococcus pneumoniae*. We investigated the LTA structure of its close relative *Streptococcus oralis*. Our analysis revealed that *S. oralis* Uo5 LTA has an overall architecture similar to pneumococcal LTA (pnLTA) and can be considered as a subtype of type IV LTA. Its structural complexity is even higher than that of pnLTA and its composition differs in number and type of carbohydrate moieties, inter-residue connectivities and especially the *P*-Cho substitution pattern. Here, we report the occurrence of a saccharide moiety substituted with two *P*-Cho residues, which is unique as yet in bacterial derived surface carbohydrates. Finally, we could link the observed important structural variations between *S. oralis* and *S. pneumoniae* LTA to the divergent enzymatic repertoire for their TA biosynthesis.

Peptidoglycan, wall teichoic acids (WTA) and lipoteichoic acids (LTA) are the major polysaccharides of the Gram-positive cell wall. The teichoic acids (TAs) of *Streptococcus pneumoniae* are unique in comparison to TAs of many other Gram-positive bacteria in several structural aspects. The composition of pneumococcal LTA (pnLTA) is the most complex of all LTAs investigated so far^1^. Pneumococcal WTA (pnWTA) and pnLTA exhibit identical structures within their repeating units (RUs)^2^, and are both decorated with phosphorylcholine (*P*-Cho) moieties^3^,^4^. This substituent is present only in cell walls of a few Gram-positive bacteria like *Streptococcus oralis*, *Streptococcus mitis*, *Streptococcus pseudopneumoniae* and *Streptococcus infantis*^5^,^6^,^7^,^8^,^9^, which are close relatives of *S. pneumoniae*. These species belong to the Mitis group of streptococci^10^, which mainly consists of commensals that reside in the oral cavity and the nasopharynx of humans. Only *S. pneumoniae* is frequently associated with diseases such as otitis media, pneumonia and meningitis, whereas e.g. *S. mitis* and *S. oralis* rarely cause disease such as endocarditis^11^,^12^. The choline-containing TAs of *S. pneumoniae* anchor choline-binding proteins (CBPs), an important family of cell surface proteins, at the cell wall and are involved in the interaction with host cells. TAs have further been described to be involved in processes like the regulation of cell wall hydrolases, the regulation of cell wall elongation and cell division, cation homoeostasis or the resistance to lysozymes and antimicrobial peptides^13^,^14^. Some CBPs—which are associated with the choline moiety of TAs by non-covalent interactions—have been identified as virulence factors specific for *S. pneumoniae*^15^,^16^. Therefore, the elucidation of structural differences between TAs of the pathogen versus those of the commensal species are of great importance.

We describe the comprehensive structural analysis of *S. oralis* Uo5 LTA, for which we combined different methodologies such as chemical degradations, high-resolution mass spectrometry (MS) as well as one- and two-dimensional, homo- and heteronuclear nuclear magnetic resonance (NMR) spectroscopy.

## Results

As starting material for our structural analysis, we used LTA of *S. oralis* Uo5Δ*cps*, which has been isolated following our previously published workflow^17^. The use of an unencapsulated variant is beneficial in terms of LTA extraction and yield. The final purification was performed with hydrophobic interaction chromatography (HIC) and fractions containing phosphate^18^ (Fig. S1A) were combined and lyophilized. In a first step, we analysed the components of *S. oralis* Uo5Δ*cps* LTA and generated defined part structures by hydrofluoric acid (HF) treatment to investigate the structure of its lipid anchor and its de-phosphorylated RU. Afterwards, we analysed the interconnection of the RUs as well as the presence and nature of phosphate-containing residues using de-*O*-acylated LTA obtained after hydrazine treatment. Additional ester-bound substituents were finally identified using the intact LTA.

### Compositional analysis

D-galactose (D-Gal*p*), D-glucose (D-Glc*p*), *N*-acetyl-D-galactosamine (D-Gal*p*NAc), 2-acetamido-4-amino-2,4,6-trideoxygalactose (AATGal*p*), ribitol (Rib-ol), glycerol (Gro), phosphate (*P*) and alanine (Ala) have been identified as constituents of *S. oralis* Uo5Δ*cps* LTA. The fatty acid analysis revealed the presence of 16:0, 16:1, 18:0 and 18:1 acids in an approximate ratio of 5.4 : 1.0 : 4.1 : 1.1, together with traces of 14:0 acid.

### Preparation and structural analysis of the lipid anchor and LTA part structures

**Figure 1:**
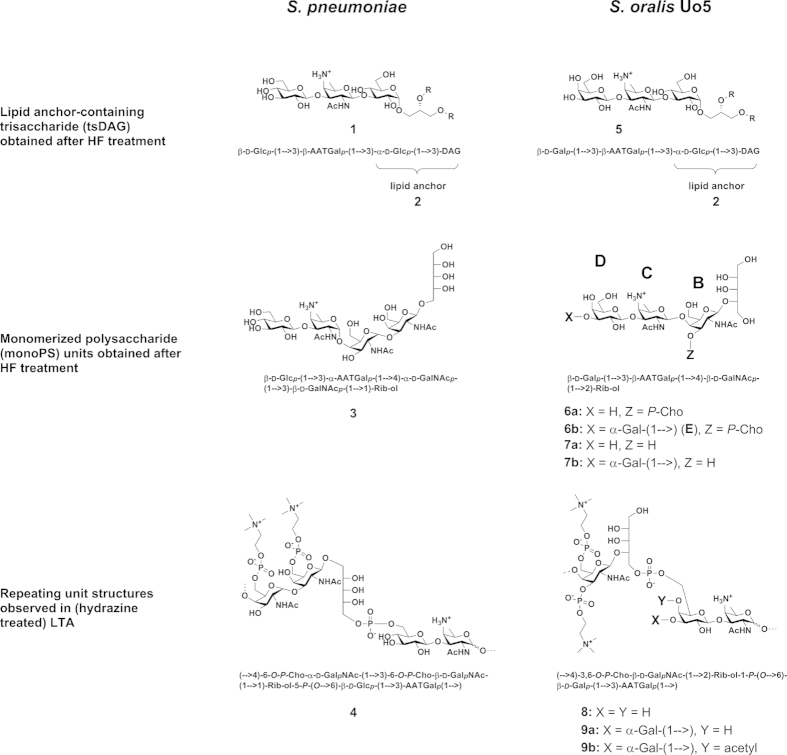
LTA part structures of *S. pneumoniae* and *S. oralis* Uo5. Compilation of the structures of the respective lipid anchor-containing trisaccharide-DAG (tsDAG; **1** and **5**, both including lipid anchor **2**; R = fatty acid) and monomerized LTA repeats (monoPS units; **3**, **6a,b** and **7a,b**) isolated after HF treatment, as well as observed repeating units in hydrazine treated or native LTA (**4**, **8** and **9a,b**).

**Figure 2:**
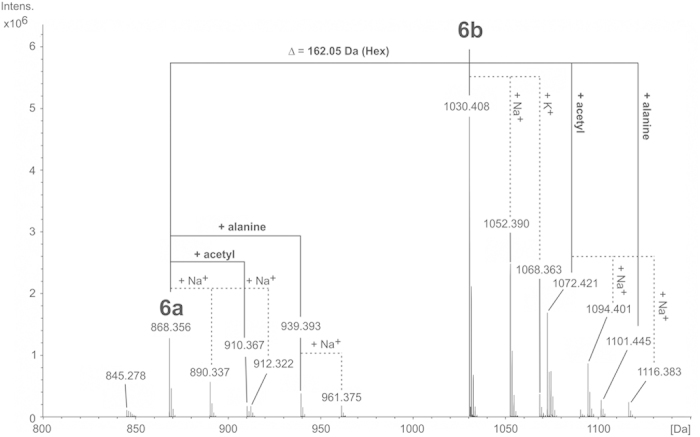
Section of the charge deconvoluted ESI-FT-ICR-MS spectra (acquired in positive ion mode) of 6a,b obtained after 2 d HF treatment of LTA of *S. oralis* Uo5∆*cps* and subsequent purification by HIC and GPC (P-10 pool 1 in Fig. S4). Besides the major molecule **6b** with a MW_found_ of 1030.408 Da (MW_calc_: 1030.403 Da), a second compound with one hexose less (**6a**; MW_found_: 868.356 Da; MW_calc_: 868.353 Da) was observed in minor amounts as well. For both molecules, variants with a bound alanine or acetyl residue could be identified with very small signal intensity.

**Figure 3:**
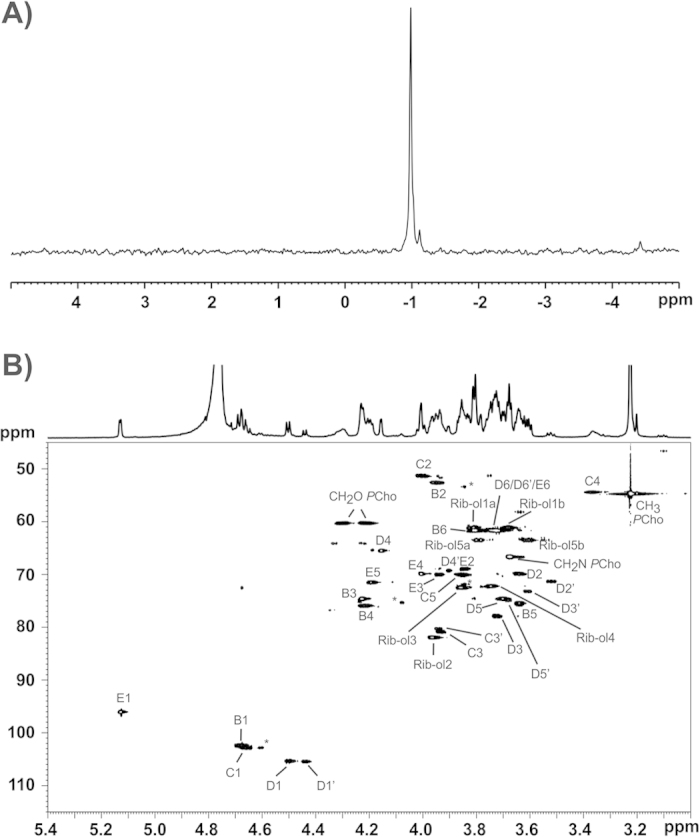
NMR analysis of 6a,b obtained after 2 d HF treatment of LTA of *S. oralis* Uo5∆*cps* and subsequent purification by HIC and GPC (P-10 pool 1 in Fig. S4; mass spectrum shown in Fig. 2). (**A**) Section (δ_P_ 5-(−5)) of the ^31^P NMR. (**B**) The respective ^1^H,^13^C-HSQC NMR spectrum (δ_H_ 5.40–3.00; δ_C_ 115–45) including assignment of signals. The presence of two molecular species—already observed in the mass spectrometrical analysis—is clearly visible and can be assigned to the absence or presence of the α-Gal moiety (E). Signals labeled with * originate from **7a,b**, which are present in small amount. All NMR chemical shift data of **6a,b** are listed in Table S3.

To elucidate the nature of the lipid anchor we treated the isolated LTA with 48% HF for two days at 4 °C. This usually cleaves all phosphodiester bonds and leads to the formation of monomerized polysaccharide (monoPS) units as well as a diacyl-glycerol (DAG) with all carbohydrate residues up to the first phosphate in the TA chain. In *S. pneumoniae* this procedure results in the formation of **1**^19^ (Fig. 1), whereas only **2** (Fig. 1) represents the biological lipid anchor. The other residues belong to the first RU of the pnLTA^17^,^20^. Therefore, **1** has to be considered as a lipid anchor-containing trisaccharide-DAG (tsDAG). Additionally, the monoPS units generated this way cannot be considered as the de-phosphorylated carbohydrate backbone of the biological RU. For pnLTA HF treatment leads mainly to *pseudo*-pentasaccharide **3**^19^,^20^ (Fig. 1), whereas the biological RU of pnLTA corresponds to **4**^17^,^20^ (Fig. 1). The HF treated LTA of *S. oralis* Uo5Δ*cps* was applied to HIC (Fig. S1B) as described for the purification of intact LTA (Fig. S1A). At early retention time (18.5 to 33.5 min; fractions 7–11 in Fig. S1B) the monomerized LTA repeats (monoPS units; **6a**, **6b**, **7a** and **7b** in Fig. 1) and other fatty acid-free part structures were eluted. To collect the lipid anchor-containing tsDAG of *S. oralis* Uo5Δ*cps* (**5**; Fig. 1), fractions between 78.5 and 102.5 min (fractions 27–34 in Fig. S1B) were combined. The mass spectrum of this latter pool is depicted in Fig. S2. The presence of *lyso*-tsDAG (respective monoacyl variants of **5**) was interpreted as a side product caused by the long HF treatment, which is required to generate the monoPS units in sufficient yield. After 8 h HF treatment (without HIC purification), the MS analysis almost exclusively displayed signals of intact **5** (calculated masses depending on the fatty acids composition: 1076.690 Da, 1104.722 Da and 1130.737 Da; Fig. S3). However, the monoPS unit formation is insufficient under these conditions. The exact structure of the carbohydrate part of *S. oralis* Uo5Δ*cps* tsDAG (**5**; Fig. 1) was analyzed by NMR (Table S1) and was similar to the pneumococcal tsDAG (**1**), but with a β-D-Gal*p*-moiety instead of the β-D-Glc*p*-moiety. The combined and lyophilized fractions of the early eluate of the HIC purification (fractions 7–11; Fig. S1B) were subsequently submitted to gel permeation chromatography (Bio-Gel P-10). A section of the chromatogram is shown in Fig. S4. All pools have been investigated separately by MS, with only P-10 pools 1 and 2 containing LTA derived polysaccharides. The completely dephosphorylated monoPS units **7a** and **7b** were identified in P-10 pool 2 (Fig. S4), the corresponding mass spectrum is shown in Fig. S5 and the assigned peaks are listed in Table S2. Very interestingly, in P-10 pool 1 (Fig. S4) monoPS units **6a** and **6b** (Fig. 1) with one *P*-Cho residue still present were identified. The corresponding mass spectrum is shown in Fig. 2 and the assigned peaks are listed in Table S2. The respective ^31^P NMR spectrum of P-10 pool 1 (Fig. 3A) provides proof for a homogeneous preparation comprising one specific *P*-Cho residue that has not been cleaved off. A section of the ^1^H,^13^C-HSQC NMR spectrum is depicted in Fig. 3B, the ^1^H and ^13^C NMR chemical shift data for **6a** and **6b** are summarized in Table S3. Some minor signals (labeled with * in Fig. 3B) are originating from **7a** and **7b**, mainly eluting in P-10 pool 2 (Fig. S4). Integration of suitable ^1^H NMR signals revealed a ratio of **6a** : **6b** of approximately 0.3 : 0.7. This result was in good agreement with the MS analysis, in which these two forms have been observed as well (Fig. 2), with **6b** as the predominant species. A similar ratio has been observed for **7a** and **7b** (Fig. S5) as well. Furthermore, an additional acetyl or alanine group has been observed, whereas the acetyl substitution seemed to be present predominantly in the molecules containing two galactosyl residues (**6b**, **7b**). However, since HF treatment can cleave off these substituents and acidic catalyzed rearrangements are possible, the presence and position of these residues has to be analysed using native LTA and will be discussed later.

### Hydrazine treatment and MS analysis

**Figure 4:**
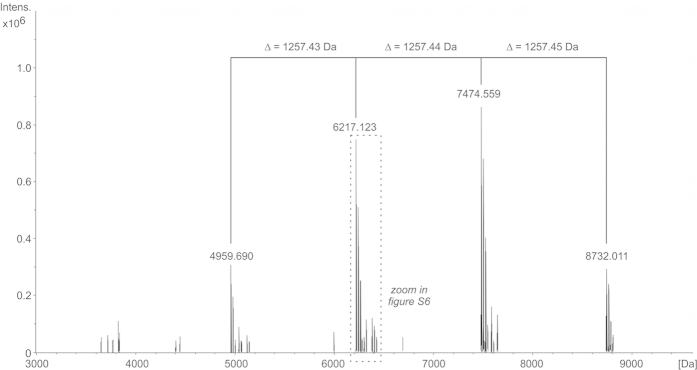
Section of the charge deconvoluted the ESI-FT-ICR-MS spectra (acquired in negative ion mode) of hydrazine-treated LTA of *S. oralis* Uo5*∆cps* (10). The monoisotopic mass of 4959.69 Da corresponds to **10** with n = 2 (structure shown in Fig. 5A), the assigned higher masses to respective molecules with longer chains (6217.123 Da (n = 3); 7474.559 Da (n = 4); 8732.011 Da (n = 5)).

**Figure 5:**
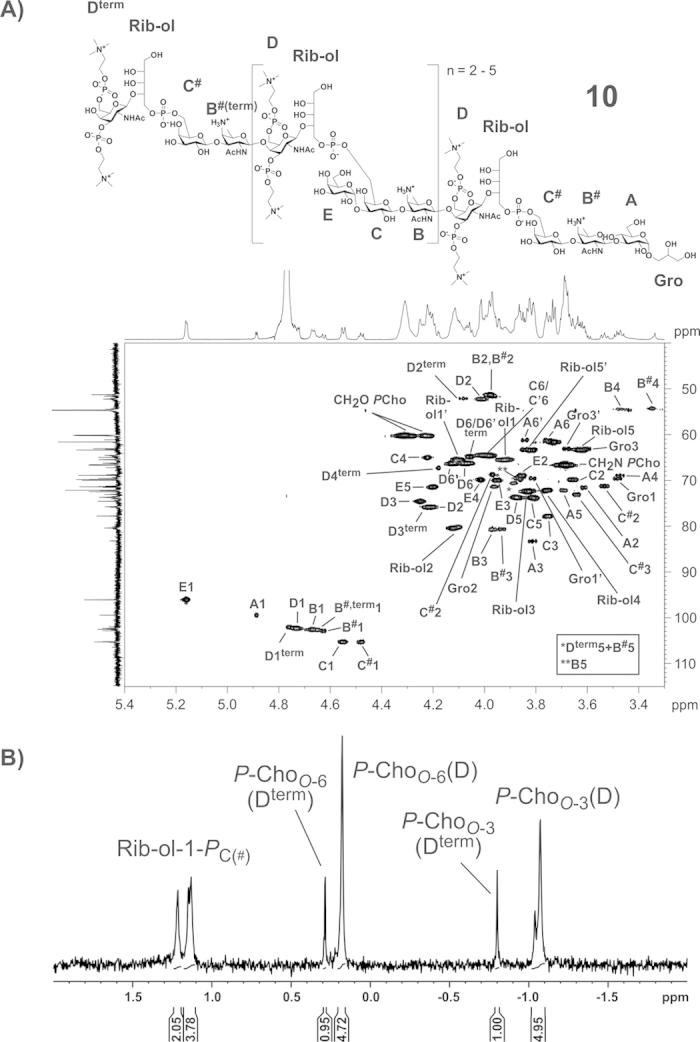
NMR analysis of 10 (mass spectrum shown in **Fig.** 4). (**A**) Section (δ_H_ 5.40–3.30; δ_C_ 115–40) of the ^1^H,^13^C-HSQC NMR spectrum including structure and assignment of signals. (**B**) Section (δ_P_ 2-(−2)) of the ^31^P NMR including assignment of signals. All NMR chemical shift data of **10** are listed in Table 1.

We have recently shown that hydrazine treatment of pneumococcal LTA^17^ can be employed to reduce the structural heterogeneity caused by differing substitution pattern of fatty acids, alanine and other ester bound residues. That has two major advantages: 1) less complex MS spectra; 2) NMR spectra show a significant higher resolution due to the absence of aggregates formed due to the lipophilic nature of LTA molecules. The mass spectrum of hydrazine treated (de-*O*-acylated) LTA of *S. oralis* Uo5*Δcps* (**10**) is shown in Fig. 4, the corresponding structure is depicted in Fig. 5A. The monoisotopic mass of 4959.69 Da in Fig. 4 corresponds to **10** (Fig. 5A) with n = 2 (4959.69 Da = Gro + Glc + 2 × RU **8** + 2 × RU **9a**). In line with the observed ratios of **6a** : **6b** and **7a** : **7b**, larger LTA molecules were detected that comprised additional RUs of structure **9a**, which can be recognized based on the specific mass difference of 1257.4 Da. Consequently, we assigned the signals at 6217.123 Da (n = 3), 7474.559 Da (n = 4) and 8732.011 Da (n = 5) to the respective LTA molecules with longer chains (n is equivalent to the number of RU with structure **9a**). The fact, that within **5** only an *O*-3-unsubstituted β-Gal has been observed (Fig. S2, Table S1), clearly reveals that the first biological RU must correspond to **8** (Fig. 1). In Fig. S6 the indicated section of Fig. 4 is enlarged, which shows a minor population of LTA molecules with five RUs comprising only one RU that lacks the α-Gal substituent (**10** with n = 3; 1 RU corresponds to **8** and 4 RU to **9a**). This structural variant has also been identified in LTA molecules with four or six RUs, respectively.

### NMR analysis of the hydrazine treated LTA (**10**)

A section of the ^1^H,^13^C HSQC NMR of **10** including the assignment of signals is depicted in Fig. 5A, the respective ^31^P NMR spectrum is shown in Fig. 5B. The detailed NMR chemical shift data of **10** are listed in Table 1. The anomeric region of the ^1^H,^13^C HSQC NMR shown in Fig. 5A reflects the complexity of the *S. oralis* Uo5 LTA. The anomeric signal of the *O*-3-substituted β-Gal (C1) can be found at δ_H_ 4.55/δ_C_ 105.3, whereas the two signals of *O*-3-unsubstituted β-Gal moieties (C^#^1) are slightly shifted in the proton spectra to δ_H_ 4.49/4.48. The respective ^13^C shifts remain comparable (δ_C_ 105.4/105.2). These two signals for C^#^1 are present due to a slightly different chemical surrounding (presence in the RU vs. in the proximity of the lipid anchor). The different β-Gal-moieties imply diversity in the AATGal-moieties as well (B/B^#^). Additionally, the terminal β-GalNAc (D^term^) can be distinguished from the β-GalNAc within the RUs (D). The signals for the two *P*-Cho-substituents of the terminal β-GalNAc (D^term^) can be differentiated in the ^31^P NMR (Fig. 5B) resulting from the ones of the comparable residues bound to the β-GalNAc placed in the RU (D) very well, since both of them are significantly shifted. Two-dimensional ^1^H,^31^P-HMQC and ^1^H,^1^H-COSY NMR experiments revealed that the *S. oralis* Uo5 LTA contains 1,2-linked ribitol moieties, in contrast to the *S. pneumoniae* LTA, which contains 1,5-linked ribitol^17^,^19^,^21^. Moreover, the ^13^C NMR chemical shift data for the ribitol moiety in **10** (Table 1) as well as in **6a**,**b** (Table S3) match very well with described ^13^C NMR values for such an 1,2-linked ribitol moiety as discovered in WTA of *Listeria monocytogenes* serotype 6^22^.

### Analysis of the native LTA

**Figure 6:**
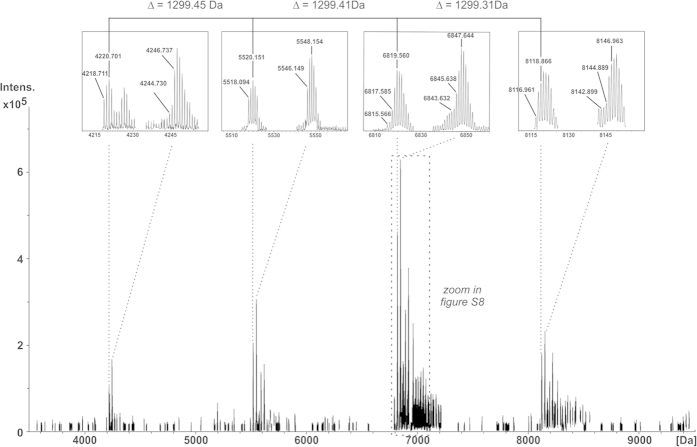
Section of the charge deconvoluted ESI-FT-ICR-MS spectra (acquired in negative ion mode) of *S. oralis* Uo5∆*cps* LTA. Repeating units (RUs) were detected with an average mass of 1299.4 Da. The RU size was determined on LTA molecules comprising a fatty acid composition with 32 carbon atoms and full saturation. The isolated LTA fraction comprised a complex mixture of molecular species as depicted in Fig. S8 and assigned molecules are summarized in Table S4.

Finally, we investigated the native *S. oralis* Uo5Δ*cps* LTA by NMR and mass spectrometry. A representative ^1^H NMR spectrum of this LTA is shown in Fig. S7. Here, the alanine-residues—which have been cleaved off during hydrazine treatment, but were present in the monoPS units after HF treatment—can be identified by their methyl group at δ_H_ 1.60 (δ_C_ 15.9). The exact position of this alanine-substitution could not be determined due to its minor occurrence. Most interestingly, another substituent that had been cleaved off during hydrazine treatment could be identified. The signal at δ_H_ 5.58 (δ_C_ 66.8) was assigned to the H-4 of β-Gal*p*, which was further substituted with an acetyl substituent at the hydroxy group at C-4, causing the shift of the signal for H-4 in the ^1^H NMR. MS analysis of the native LTA (Fig. 6) indicated that all RUs containing the α-Gal*p* substituent also bear this acetyl group, since the mass of the RU in the native LTA is 1299.4 Da (corresponding to **9b**; Fig. 1). The mass difference of 42.0 Da compared to RU **9a** present in **10** (Fig. 4) matches an additional acetyl group. The molecules with a combined fatty acid composition of 32:0 have been chosen in Fig. 6 for calculations of the RU size, which exemplifies the composition of all LTA molecules (Table S4). Furthermore, we recognized a variation in the number of alanine residue per LTA of zero to three by this MS analysis (representative magnified section in Fig. S8). We identified 32 molecular species within the LTA fraction that are summarized in Table S4.

**Figure 7:**
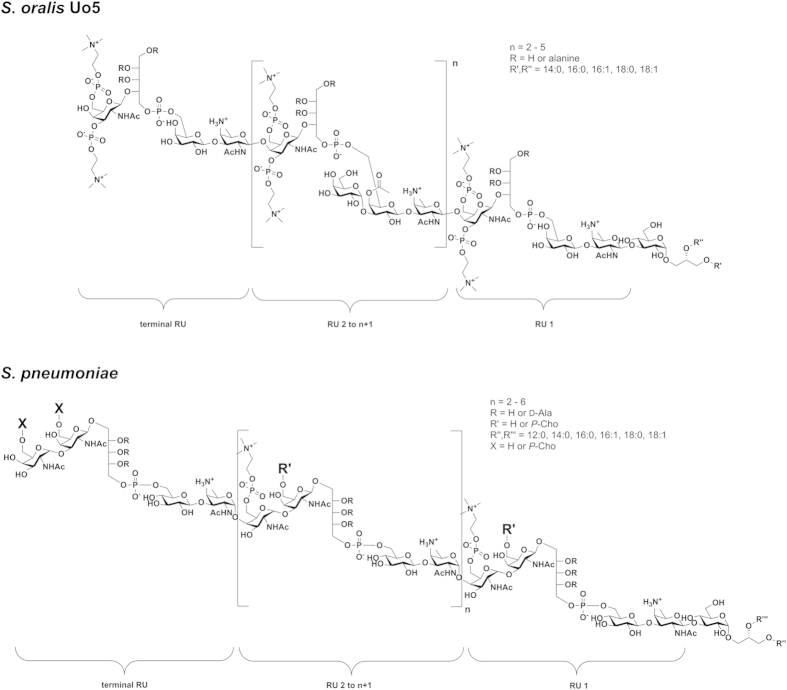
Structure for *S. oralis* Uo5 LTA compared to *S. pneumoniae* LTA. In the *S. oralis* Uo5 LTA, the repeating units (RUs) are composed of a *pseudo*-tetrasaccharide chain ((→ 4)-[3,6-*O*-di-*P*-Cho]-β-D-Gal*p*NAc-(1 → 2)-Rib-ol-(1-*P* → 6)-(β-D-Gal*p*-(1 → 3)-β-AATGal*p*-(1 →) (**8** in Fig. 1)), RU 2 to n + 1 bear an additional α-1-linked D-Gal*p* moiety at *O*-3 and an acetyl residue at *O*-4 of the β-D-Gal*p* (**9b** in Fig. 1). Hydroxyl groups of the ribitol-1-*P* can be partially substituted with alanine. The chain length is 4 to 7 RUs. In *S. pneumoniae* LTA^23^, all RUs comprise the *pseudo*-pentasaccharide ((→ 4)-6-*O*-*P*-Cho-α-D-Gal*p*NAc-(1 → 3)-6-*O*-*P*-Cho-β-D-Gal*p*NAc-(1 → 1)-Rib-ol-5-*P*-(*O* → 6)-β-D-Glc*p*-(1 → 3)-AATGal*p*-(1 →)) (**4** in Fig. 1), the terminal RU can occur with or without 6-*O*-*P*-Cho-substitution (X = H or *P*-Cho). Known strains with the content of only one *P*-Cho per RU lack the *P*-Cho at β-D-Gal*p*NAc (R′ = H). Hydroxyl groups of Rib-ol-5-*P* can be partially substituted with D-Ala. The chain length of pnLTA is 4 to 8 RUs in general. Notably, in pnLTA the first repeating unit is β-1-linked via the AATGal*p* to the lipid anchor, all other RUs are α-1-linked to the previous one. In *S. oralis* Uo5 LTA all AATGal*p* residues are β-configurated.

Finally, combination of the results from the NMR spectroscopy and MS of native as well as chemically degraded LTA (part structures) allowed us to resolve the structure of *S. oralis* Uo5 LTA, which is depicted in comparison to the pneumococcal LTA^23^ in Fig. 7. The glycolipid anchor of the *S. oralis* Uo5 LTA is the same as in the pneumococcal LTA: α-D-Glc*p*(1 → 3)-DAG (**2**; Fig. 1). The subsequent repeating unit (RU 1) consists of the *pseudo*-tetrasaccharide **8** (Fig. 1). The following RUs (RU 2 to n + 1) have a structurally identical chain but are further substituted at the β-D-Gal*p* with an α-D-Gal*p*-(1 → )-moiety in position *O*-3 and an acetyl residue in position *O*-4 (**9b**; Fig. 1). Our analysis revealed that in addition to RU 1 another RU in the molecule lacks the α-D-Gal*p* and acetyl substitution (Figs 2, 3B and 5A). Based on our analysis, we conclude that this is the terminal RU, even if the final proof for this has yet to come. Our attempts to address this question by MS/MS-experiments or the investigation of other LTA part structures (e.g. after Smith degradation) failed so far.

### Implication of the structural analysis for understanding the type IV LTA biosynthesis

**Figure 8:**
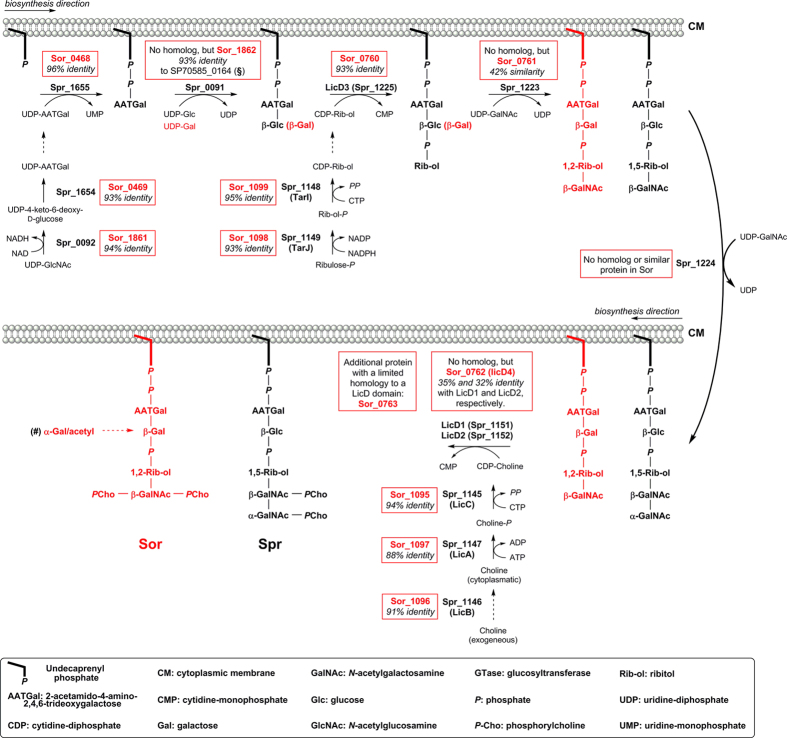
Biosynthesis pathway for the teichoic acid repeating unit in *S. pneumoniae* (black; Spr numbers represents *S. pneumoniae* R6 proteins) highlighting similar or homologous genes in *S. oralis* (red; Sor numbers represents *S. oralis* Uo5 proteins). The biosynthesis pathway of TAs in *S. pneumoniae* R6 and identity to *S. oralis* Uo5 proteins is according to reference^24^. (§) The gene encoding this protein was identified in the genome of an *S. pneumoniae* serotype 5 strain, which has been shown to incorporate Gal instead of Glc into its WTA^25^. (#) Neither the genes encoding the proteins responsible for the addition of the α-Gal and acetyl residue to the β-Gal, nor the time point of these modifications in the biosynthesis pathway have been identified so far.

Unlike other Gram-positive bacteria, the TAs in *S. pneumoniae*, *S. oralis* and *S. mitis* share a common biosynthetic pathway, which has been bioinformatically analysed recently^24^. These investigations indicated, that *S. oralis* Uo5 LTA has the same linker as *S. pneumoniae* LTA (**2**; Fig. 1)^24^, what we confirmed by structural analysis (Table S1). The biosynthetic steps of TA repeating unit formation in *S. oralis* Uo5 are summarized in Fig. 8 in comparison with the genetic machinery present in *S. pneumoniae*. Hereby, the close link between the deviating enzymatic repertoire for TA biosynthesis in *S. pneumoniae* R6 and *S. oralis* Uo5 and therefore varying structural elements in their TAs is demonstrated. The first sugar moiety of the biological TA repeating unit in *S. oralis* Uo5 is an AATGal as in *S. pneumoniae*. All enzymes required for the synthesis of its activated precursor (Sor_1861, Sor_0469) as well as for the transfer to the undecaprenyl-phosphate linker in the membrane (Sor_0468) are present in the *S. oralis* Uo5 genome with high identity (93–96%) to the respective enzymes in *S. pneumoniae* strain R6 (Spr_0092, Spr_1654, Spr_1655)^24^. The second carbohydrate moiety in pnTAs is a β-Glc*p*, which is transferred by Spr_0091, whereas our structural analysis revealed a β-Gal*p* being present in the *S. oralis* Uo5 LTA at this position. This β-Gal*p* is incorporated by the glycosyl transferase Sor_1862, which has 93% identity to SP70585_0164, representing a glycosyl transferase which transfers a β-Gal*p* to the AATGal moiety in TAs of a pneumococcal serotype 5 strain^24^,^25^. Subsequently, a phosphate-bridged ribitol is incorporated by LicD3. *S. oralis* LicD3 (Sor_0760) has 93% identity with the pneumococcal LicD3 (Spr_1225)^24^. In contrast to pnTAs, in which the structural element α-Gal*p*NAc-(1 → 3)-β-Gal*p*NAc-(1 → ) is present at the ribitol, only a β-Gal*p*NAc-(1 → ) moiety has been identified in the *S. oralis* Uo5 TA repeating unit. The glycosyl transferase Sor_0761 has 42% similarity with Spr_1223, but no homolog or similar enzyme to Spr_1224 has been found in the *S. oralis* Uo5 genome^24^. Therefore, it is most likely that Spr_1223 incorporates the β-Gal*p*NAc and Spr_1224 the α-Gal*p*NAc into pnTAs, respectively. Notably, the ribitol-phosphate in pnTAs is 1,5-linked, whereas a 1,2-linkage is present in *S. oralis* Uo5 LTA. This is in accordance with the low similarity of Sor_0761 and Spr_1223. The pneumococcal choline uptake system consisting of LicA, B, and C is present in *S. oralis* Uo5 with high identity (88–94%) as well. In *S. pneumoniae*, *P*-Cho is attached by the LicD1 (Spr_1151) and LicD2 (Spr_1152) proteins to the *O*-6-positions of the Gal*p*NAc moieties. In *S. oralis* no homologs to LicD1 and LicD2 have been identified. Instead, LicD4 (Sor_0762) is present, which contains a C-terminal region of 35% and 32% sequence identity compared to pneumococcal LicD1 and LicD2, respectively^24^. Certainly, LicD4 incorporates at least the *P*-Cho substituent at the *O*-6-position of β-Gal*p*NAc in *S. oralis* Uo5. If the *P*-Cho substituent at the *O*-3-position is incorporated by LicD4 as well has to be investigated further. Another potential candidate for this is a protein (Sor_0763) encoded downstream of *licD4* with very limited homology to a LicD domain. Neither the genes encoding the proteins responsible for the addition of the α-Gal*p* and the acetyl residue to the β-Gal*p*, nor the time point of these modifications in the biosynthesis pathway have been identified so far.

## Discussion

The present study provides the first structural elucidation of an *S. oralis* LTA. For our analysis, we generated an unencapsulated variant of strain Uo5 (*S. oralis* Uo5Δ*cps*). The structure of the isolated LTA is of higher complexity than in any other reported teichoic acid. Due to a close genetic relationship to *S. pneumoniae*, a ribitol-phosphate containing type IV LTA^1^ was anticipated. Combined structural analysis by NMR spectroscopy and mass spectrometry of natural as well as chemically generated LTA part structures allowed us to resolve its structure (Fig. 7). We could show that the *S. oralis* Uo5 LTA possesses an overall architecture similar to the pnLTA, but differs with regard to the number of Gal*p*NAc moieties, inter-residue connectivities, positions of the *P*-Cho substituents and has additional, off-stoichiometric branch substituents. With help of the detailed structural analysis, we could improve the functional assignments for genes involved in the *S. oralis* Uo5 TA biosynthesis in comparison to those identified in *S. pneumoniae* (Fig. 8). This led in turn to a more specific assignment of enzyme functionalities in *S. pneumoniae* as well, since it appears now obvious that Spr_1223 incorporates the β-Gal*p*NAc and Spr_1224 the α-Gal*p*NAc into pnTAs.

Our results indicate that in *S. oralis* Uo5 additional modifications take place, that—causally determined by the different TA structure—do not happen in *S. pneumoniae*. As shown in Fig. S6, we observed a small portion of LTA molecules comprising only one RU of structure **8** (all other RU correspond to **9b**), whereas the majority of LTA molecules contained two of them. Presumably, these modifications (removal of one α-Gal*p* moiety and one acetyl residue) of the TA chain occur after transfer through the cell membrane and onto the glycolipid anchor. In *S. pneumoniae*, the terminal RU is modified under certain conditions, in this case with respect to its *P*-Cho content^17^. Besides the TAs of *S. pneumoniae* and *S. oralis*, the modification of glycans with *P*-Cho residues is known for some capsular polysaccharides of *S. pneumoniae* as well as for lipopolysaccharides of a few organisms, especially *H. influenzae*. These *P*-Cho moieties have been found to be attached to the *O*-2, *O*-3, *O*-4 or *O*-6 positions of different hexoses as well as to *O*-7 of a heptose^8^. However, to the best of our knowledge, this is the first report of a bacterial derived glyco(lipid) structure containing a sugar residue with two *P*-Cho substituents. Therefore, the identification and investigation of the responsible *P*-Cho transferring enzymes will be of interest. Further studies have to show, whether the positions of the *P*-Cho residues have an influence on the attachment and the number of CBPs in *S. oralis* compared to *S. pneumoniae* and *S. mitis*. Several physiological relevant CBPs are highly conserved between *S. pneumoniae* and *S. mitis*^26^, being in line with the structurally identical TA repeating unit discovered for *S. mitis* strain SK137^7^. This is further corroborated by the bioinformatic analysis of the genome of *S. mitis* strain B6, which predicted a very similar structure compared to the pneumococcal TA, merely with the exchange of the β-D-Glc*p* by a galactose^24^. In contrast, only a small number of CBPs has been identified in *S. oralis* Uo5, including those playing a principal role in cell physiology: LytB, LytC, CbpF, and two paralogues of CbpD^27^,^28^. As discovered in *S. pneumoniae*^17^,^29^, the LTA of *S. oralis* Uo5 was found to be partially substituted with alanine at its ribitol moieties. D-alanine substituents in the repeating units of LTA polymers, both in *S. pneumoniae* and in *S. aureus*, have been thought to play essential roles in their biological activity, especially with respect to their pro-inflammatory potency and activation of the Toll-like receptor 2 (TLR2)^30^,^31^,^32^. Meanwhile it has been demonstrated that lipoproteins (LPs) are the predominant TLR2 stimuli in LTA preparations of *S. aureus* and *S. pneumoniae*, respectively, and not the LTA itself^17^,^33^,^34^. Another study suggested that, like in many other low-G + C Gram-positive bacteria, teichoic acids of *S. pneumoniae* contain D-alanine residues in order to protect the bacterium against the actions of cationic antimicrobial peptides^35^. Some of the players involved in the TA repeating unit biosynthesis of *S. oralis* Uo5 remained elusive so far and seem to be unique for *S. oralis*. Particularly, the enzymes for the addition and potential removing of the α-D-Gal*p*-(1 → )-moiety in position *O*-3 and the acetyl residue in position *O*-4 of the β-D-Gal*p* have to be identified. All together, the detailed knowledge of these complex structural features of *S. oralis* LTA will help to decipher the molecular requirements for the interaction of TAs and CBPs in human pathogens like *S. oralis* and *S. pneumoniae*.

## Methods

### Bacterial strain and growth conditions

Bacteria were grown at 37 °C without aeration in C medium^36^ supplemented with 0.1% yeast extract (C + Y medium) or on D-agar plates^37^ supplemented with 3% defibrinated sheep blood. The growth of bacteria in liquid culture was monitored by measuring the optical density at 600 nm.

### Genetic transformation and DNA techniques

The transformation of *S. oralis* Uo5 for strain construction was carried out as described previously for *S. pneumoniae* strains^38^. Transformants were selected by plating on D-agar supplemented with 3% defibrinated sheep blood and 200 μg/ml kanamycin.

All DNA manipulations were performed using standard procedures^39^. DNA was amplified with iProof high-fidelity DNA polymerase (Bio-Rad Laboratories).

### Construction of **S. oralis** Uo5Δ**cps**

To construct the Uo5Δ*cps* mutant, in which the capsule locus (genes from *sor_1646* to *sor_1661*) was completely deleted, a kanamycin cassette flanked by DNA corresponding to the upstream and downstream regions of the *cps* locus was designed. A DNA fragment corresponding to the upstream region of *cps* was amplified by PCR from Uo5 strain using the primers U05_cps_pcr1_f and U05_cps_pcr1_r, whereas downstream fragment of *cps* was amplified using the primers U05_cps_pcr3_f and U05_cps_pcr3_r. A 1010-bp fragment containing kanamycin resistance gene *aphIII*, including the promotor and terminator regions, was amplified from plasmid pMP1 DNA using the primers U05_cps_pcr2_f and U05_cps_pcr2_r. The three PCR fragments were purified and used as a template in a fusion PCR using primers U05_cps_pcr1_f and U05_cps_pcr3_r. The resulting PCR fragment was purified und transformed into Uo5 competent cells. The correct integration was verified by PCR and sequencing. In *S. oralis*, insertion of the kanamycin cassette produced a 14,537-bp deletion. The oligonucleotides used for construction of these DNA fragments were obtained from Eurofins MWG Operon and are listed in Table S5.

### Cell wall preparation

*S. oralis* Uo5Δ*cps* was grown in 5-liter batches in C + Y medium. Bacterial cells were harvested at an OD_600_ of appr. 1.0 by centrifugation (7,500 × *g*, 10 min) at room temperature. The cell pellet was washed with citric buffer (50 mM, pH 4.7) and resuspended in citric buffer containing 4% sodium dodecyl sulfate. The cell suspension was incubated for 20 min at 100 °C, stored at −80 °C and lyophilized subsequently.

### LTA extraction and purification

LTA extraction and purification by hydrophobic interaction chromatography (HIC) was performed as described for pnLTA^17^. Cells were resuspended in distilled water prior to extraction, thus restoring the concentration of citric buffer (50 mM, pH 4.7) and SDS (4%). Cells were disrupted in a cell homogenizer (Vibrogen Cell Mill VI 6, Edmund Bühler GmbH) with 0.1 mm glass beads (Carl Roth GmbH). Afterwards, glass beads were removed by centrifugation (2,000 × *g*, 10 min, 20 °C) and washed twice with citric buffer. To the combined supernatants SDS (20% solution) was added to have a final concentration of 4%. This solution was incubated for 30 min at 100 °C and centrifuged (30,000 × *g*, 15 min, 4 °C) subsequently. The pellet was washed four times with citric buffer, all supernatants combined and lyophilized. The resulting solid was washed SDS-free with ethanol (10,650 × *g*, 15 min, 20 °C). The resulting sediment was resuspended in citric buffer and extracted with an equal volume of butan-1-ol (Merck) for 30 min at RT under vigorous stirring. Phases were separated at 2,100 × *g* and 4 °C for 15 min. The aqueous phase was removed and the extraction procedure was repeated twice. The combined LTA containing water phases were lyophilized prior to dialysis for 5 d at 4 °C against 50 mM ammonium acetate buffer (pH 4.7; 3.5 kDa cut-off; buffer change usually every 24 h). A final lyophilization leads to crude LTA which is further purified by hydrophobic-interaction chromatography (HIC) performed on a HiPrep octyl-sepharose column (GE Healthcare; 16 × 100 mm, bed volume 20 ml). The crude LTA material was dissolved in less starting buffer (15% propan-1-ol (Merck) in 0.1 M ammonium acetate (pH 4.7)) as possible, centrifuged (13,000 × *g*, 5 min, 20 °C) and the obtained supernatant was subjected to HIC using a linear gradient from 15% to 60% propan-1-ol in 0.1 M ammonium acetate (pH 4.7). LTA-containing fractions were identified by a photometric phosphate test^18^ and appropriate phosphate-containing fractions were combined (Fig. S1A), lyophilized and repeatedly washed with water on freeze-drying to remove residual buffer. The extraction of 10 l bacterial culture yielded 21.7 mg purified LTA.

### Hydrazine-treatment of LTA

LTA preparations were dissolved 5 μg/μl in anhydrous hydrazine (N_2_H_4_; ICN Biomedicals) and then incubated for 1 h at 37 °C and 100–150 rpm (orbital shaker). The reaction was quenched by adding the same volume of acetone and dried under a stream of nitrogen. The latter step was repeated twice and the crude *O*-deacylated LTAs were purified by gel permeation chromatography (GPC) on a column using Bio-Gel P-10 (45–90 μm, BioRad; column size: 1.5 × 120 cm; buffer: 150 mM ammonium acetate (pH 4.7)) and Toyopearl TSK-40S (Tosoh Bioscience; column size: 2.5 × 50 cm; buffer: 50 mM pyridine/acetic acid/water (8/20/2000 v/v/v)) in a sequential order^17^.

### Preparation of the lipid anchor and monomerized polysaccharides

To generate the lipid anchor as well as TA derived polysaccharides, LTA was treated with 48% hydrofluoric acid (HF) at 4 °C for 2 d (20 μl HF/mg LTA). After drying under a stream of nitrogen at room temperature, the resulting residue was resuspended in water and lyophilized. With this material a purification by HIC as used for the purification of native LTA was performed (Fig. S1B). The polysaccharides eluted in the void volume, the lipid anchor in a comparable retention time period as the natural LTA. After lyophilization, a gel permeation chromatography (GPC) on a column using Bio-Gel P-10 (45–90 μm, BioRad; column size: 1.5 × 120 cm; buffer: 150 mM ammonium acetate (pH 4.7)) was performed with the polysaccharide containing material (Fig. S4); polysaccharides were monitored by a Knauer differential refractometer.

### Analytical chemistry

Compositional analysis of the LTA (50–200 μg) was done by gas-liquid chromatography–mass spectrometry (GLC–MS) after methanolysis (0.5 M HCl/CH_3_OH, 30 min, 85 °C) and per-*O*-acetylation (pyridine/Ac_2_O (1:1, v/v), 85 °C, 15 min) with or without prior HF treatment (48% HF, 4 °C, 3 d). The configuration of the aldopyranosides was determined by GLC–MS of acetylated (*S*)-2-butyl glycosides in comparison to authentic standards^40^. Fatty acids were recovered from the chloroform phase after alkaline hydrolysis of LTA (1 M NaOH in 50% aqueous CH_3_OH, 2 h, 85 °C) and detected as fatty acid methyl esters (diazomethane in CHCl_3_/CH_3_OH (95:5, v/v)), 5 min, room temperature). For quantification, *n*-heptadecanoic acid (17:0; Sigma) was used as internal standard. Reported data for fatty acid ratios are the mean of five independent hydrolysis of the same LTA batch. All GLC–MS analyses were performed on an Agilent Technologies 6890N gas chromatograph coupled to a 5975 inert XL Mass Selective Detector. A 30-m HP-5MS column (Hewlett-Packard) was used and the applied temperature gradient started at 70 °C (1.5 min), was increased linearly with 60 °C/min to 150 °C, kept there for 3 min, and then increased linearly with 5 °C/min to 320 °C.

### Mass spectrometry

For Electrospray Ionization Fourier-Transform Ion Cyclotron Resonance Mass Spectrometry (ESI-FT-ICR-MS) analysis a 7 Tesla APEX Qe instrument (Bruker Daltonics, Bremen, Germany) has been used. For measurement in the negative-ion mode samples were dissolved in a water/propan-2-ol/7 M triethylamine/acetic acid mixture (50:50:0.06:0.02, v/v/v/v), for the positive-ion mode a water/propan-2-ol/30 mM ammonium acetate/acetic acid mixture (15:15:1:0.04, v/v/v/v) was used. Spectra were acquired in broadband acquisition mode either with micro-ESI using a flow rate of 2 μl/min with a capillary voltage set to −3.8 kV or with nano-ESI using the Triversa Nanomate (Advion, USA) as ion source with a spray voltage set to −1.1 kV. The mass scale was externally calibrated with glycolipids of known structure, all spectra were smoothed and charge deconvoluted. The given mass numbers refer to the monoisotopic mass of the neutral molecules.

### NMR spectroscopy

Deuterated solvents were purchased from Deutero GmbH (Kastellaun, Germany). NMR spectroscopic measurements were performed in D_2_O at indicated temperatures on a Bruker Avance^III^ 700 MHz (equipped with an inverse 5 mm quadruple-resonance Z-grad cryoprobe). Acetone was used as an external standard for calibration of ^1^H (δ_H_ = 2.225) and ^13^C (δ_C_ = 30.89) NMR spectra^41^, 85% phosphoric acid was used as an external standard for calibration of ^31^P NMR spectra (δ_P_ = 0.0) at the respective temperatures. Analysis of **5** was performed in CD_3_OD, spectra were calibrated using the residual solvent peak (δ_H_ = 3.31, δ_C_ = 49.0)^41^. All data were acquired and processed using Bruker TOPSPIN V 3.0 or 3.1. ^1^H NMR assignments were confirmed by 2D ^1^H,^1^H-COSY and -TOCSY experiments, ^13^C NMR assignments were indicated by 2D ^1^H,^13^C-HSQC, based on the ^1^H NMR assignments. Interresidue connectivity and further evidence for ^13^C assignment were obtained from 2D ^1^H,^13^C-HMBC and ^1^H,^13^C-HSQC-TOCSY. Connectivity of phosphate groups were assigned by 2D ^1^H,^31^P-HMQC and ^1^H,^31^P-HMQC-TOCSY.

## Additional Information

**How to cite this article**: Gisch, N. *et al.* Lipoteichoic acid of *Streptococcus oralis* Uo5: a novel biochemical structure comprising an unusual phosphorylcholine substitution pattern compared to *Streptococcus pneumoniae*. *Sci. Rep.* **5**, 16718; doi: 10.1038/srep16718 (2015).

## Supplementary Material

Supplementary Information

## Figures and Tables

**Table 1:** ^1^H (700.7 MHz), ^13^C NMR (176.2 MHz), and ^31^P NMR (283.7 MHz) chemical shift data (δ, ppm) [*J*, Hz] for *S. oralis* Uo5Δ*cps* LTA after hydrazine treatment (10).

Residue (assignment)	H-1 *C-1*	H-2 *C-2*	H-3 *C-3*	H-4 *C-4*	H-5 *C-5*	H-6 *C-6*	NAc
Glycerol-(1→ (**Gro**)	3.83–3.79*	3.62–3.58*	3.70–3.66*				
	3.50–3.46*						
	*69.5*	*71.3*	*63.1*				
→3)-α-D-Glc*p*-(1→ (**A**)	4.89 [3.6]	3.63–3.60*	3.84–3.80*	3.49–3.46*	3.71–3.67*	3.86–3.82*	
						3.77–3.74*	
	*99.4*	*71.5*	*83.2*	*68.7*	*72.2*	*61.1*	
→3)-β-AATGal*p*-(1→ (**B**^**#**^)	4.63 [8.2]	3.98–3.93*	3.95–3.91*	3.37–3.32*	3.90–3.87*	1.29 [6.4]	2.02
	*102.8*	*51.6*	*80.6*	*54.3*	*70.5*	*16.7*	*22.9*
							*175.7*
*P*→6)-β-D-Gal*p*-(1→ (**C**^**#**^)	4.49/4.48	3.56–3.51*	3.66–3.63*	3.98–3.96*	3.83–3.78*	4.03–3.96*	
	[7.7]						
	*105.4/105.2*	*71.2*	*73.1/73.0*	*68.7*	74.1–73.9*	64.7–64.5*	
→2)-ribitol-(1→ *P* (**Rib-ol**)	4.13–4.08*	4.15–4.08*	3.86–3.81*	3.78–3.73*	3.84–3.79*		
	3.94–3.90*				3.65–3.60*		
	65.6–65.4*	80.5–80.3*	*72.5*	*72.1*	*63.4*		
→4)-β-D-3,6-*O*-di-*P*-Cho-Gal*p*NAc (1→ (**D**)	4.73 [8.7]	4.04–3.98*	4.27–4.23*	4.24–4.18*	3.90–3.85*	4.15–4.11*	2.07
						4.09–4.03*	
	102.3	52.3–52.1*	74.6–74.5	75.8–75.7	73.8–73.6*	66.3–66.2	*23.2*
							*175.1*
Cho-*P*-(3-*O*→ @ **D**	4.32–4.28*	3.73–3.67*	3.24/3.23				
	4.25–4.20*						
	60.3–60.1*	66.7–66.5*	*54.6*				
Cho-*P*-(6-*O*→ @ **D**	4.34–4.28*	3.71–3.67*	3.24				
	60.3–60.1*	66.7–66.5*	*54.6*				
→3)-β-AATGal*p*-(1→ (**B**)	4.69–4.65*	4.00–3.94*	4.00–3.95*	3.50–3.44*	3.89–3.84*	1.33–1.29*	2.06
	*102.5*	*51.3*	*80.6*	*54.5*	*69.4*	*16.8*	*23.0*
							*175.6*
(E)→3,*P*→6)-β-D-Gal*p*-(1→ (**C**)	4.55 [7.7]	3.68–3.63*	3.78–3.73*	4.24–4.20*	3.83–3.78*	4.03–3.96*	
	*105.3*	*69.8*	*77.8*	*65.0*	*74.1–73.9*	*64.7–64.5*	
α-D-Gal*p*-(1→ (**E**)	5.16 [3.6]	3.88–3.84*	3.97–3.93*	4.03–4.00*	4.22–4.18*	3.74–3.71*	
	*96.1*	*68.9*	*70.0*	*69.8*	*71.4*	*61.6*	
(C^#^)→3)-β-AATGal*p*-(1→ (**B**^**#,term**^)	4.65 [8.2]	3.99–3.95*	3.99–3.95*	3.37–3.32*	3.89–3.84*	1.33–1.29*	2.07
	*102.6*	*51.4*	*80.6*	*54.7*	*70.5*	*16.8*	*22.9*
							*175.7*
β-D-3,6-*O*-di-*P*-Cho-Gal*p*NAc-(1→ (**D**^**term**^)	4.76 [8.6]	4.10–4.06^*^	4.24–4.20^*^	4.19–4.17*	3.90–3.86*	4.08–4.04*	2.09
	*102.0*	52.2–52.0*	*75.9–75.8*	*67.2*	70.6–70.5*	*64.8 [4.8]*	
							*23.3*
							*175.5*
Cho-*P*-(3-*O*→@ **D**^**term**^	4.31–4.26*	3.69–3.65*	3.22				
	4.24–4.20*						
	60.3–60.1*	66.7–66.5*	*54.6*				
Cho-*P*-(6-*O*→@ **D**^**term**^	4.35–4.31*	3.71–3.67*	3.24				
	60.3–60.1*	66.7–66.5*	*54.6*				
^**31**^**P**	*P*-1^Rib-ol^/6^C(#)^ 1.21; *P*-1^Rib-ol^/6^C^ 1.15, 1.13; *P*-6^D(term)^/CH_2_O^Cho^ 0.29; *P*-6^D^/CH_2_O^Cho^ 0.18; *P*-3^D(term)^/CH_2_O^Cho^ −0.80; *P*-3^D^/CH_2_O^Cho^ −1.04, −1.07.						

^*^non-resolved multiplet.
