# Corrigendum: Lipoteichoic acid of *Streptococcus oralis* Uo5: a novel biochemical structure comprising an unusual phosphorylcholine substitution pattern compared to *Streptococcus pneumoniae*

**DOI:** 10.1038/srep24424

**Published:** 2016-04-22

**Authors:** Nicolas Gisch, Dominik Schwudke, Simone Thomsen, Nathalie Heß, Regine Hakenbeck, Dalia Denapaite

Scientific Reports 5: Article number: 1671810.1038/srep16718; published online: 11182015; updated: 04222016

In Table 1 the ^1^H NMR resonances for glycerol (Gro) positions 2 and 3 are incorrect. In the corresponding Figure 5A the respective signals have been assigned correctly.

The correct chemical shift of Gro2 in ^1^H NMR is δ_H_ 3.97–3.94*, for Gro3′ and Gro3 δ_H_ 3.70–3.66* and δ_H_ 3.62–3.58*, respectively.

These errors do not affect the structural determination published in the paper or the interpretation of the results or the conclusions of the work.

