# Supplementary material for: Lipoteichoic acid of Streptococcus oralis Uo5: a novel biochemical structure comprising an unusual phosphorylcholine substitution pattern compared to Streptococcus pneumoniae
Source: Sci Rep. 2015 Nov 18;5:16718. doi: 10.1038/srep16718 (PMC4649388; doi:10.1038/srep16718)
Supplement: Supplementary Information [file srep16718-s1.pdf]

SUPPLEMENTARY INFORMATION

Lipoteichoic acid of *Streptococcus oralis* Uo5: a novel biochemical structure comprising an unusual phosphorylcholine substitution pattern compared to *Streptococcus pneumoniae*

Nicolas Gisch[1,*], Dominik Schwudke[1], Simone Thomsen[1], Nathalie Heß[2], Regine Hakenbeck[2], and Dalia Denapaite[2]

[1]Division of Bioanalytical Chemistry, Priority Area Infections, Research Center Borstel, Leibniz-Center for Medicine and Biosciences, Parkallee 1-40, 23845 Borstel, Germany

[2]Department of Microbiology, University of Kaiserslautern, Paul-Ehrlich Straße 24, 67663 Kaiserslautern, Germany

*Correspondence to: Nicolas Gisch, Division of Bioanalytical Chemistry, Research Center Borstel, Leibniz-Center for Medicine and Biosciences, Parkallee 4a/4c, 23845 Borstel, Germany. Email: ngisch@fz-borstel.de

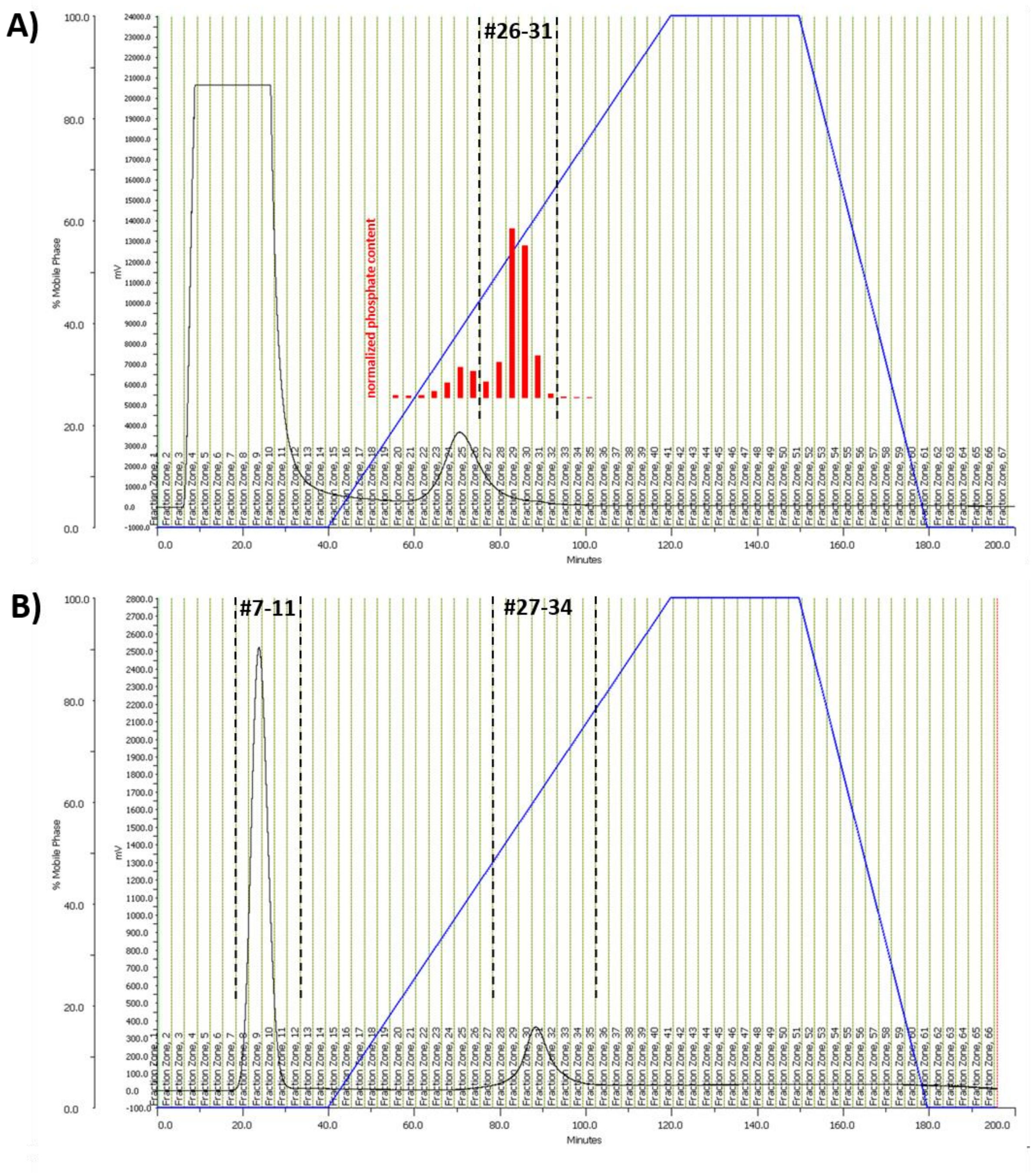

Figure S1. Representative chromatogram of the hydrophobic interaction chromatography of A) native *S. oralis* Uo5Δ*cps* LTA including normalized visualization of the phosphate content of selected fractions and B) *S. oralis* Uo5Δ*cps* LTA after 2 d HF treatment (UV detection in A) and B) at $\lambda = 254$ nm). In A) combining of fractions is based on the phosphate content, fractions #26-31 contain the LTA. The major UV absorption observed in fractions #21-25 is caused by other, non-LTA species, such as lipopeptides. In B) combining of fractions is based on the UV absorption, since HF treatment removes the phosphate groups (**Attention**: consider the 10-fold less mV-scale in B) compared to A)). Fractions #7-11 contain among other things the monoPS units **6a,b** and **7a,b**, their further purification is shown in Fig. S4. Fractions #27-34 contain the tsDAG **5**.

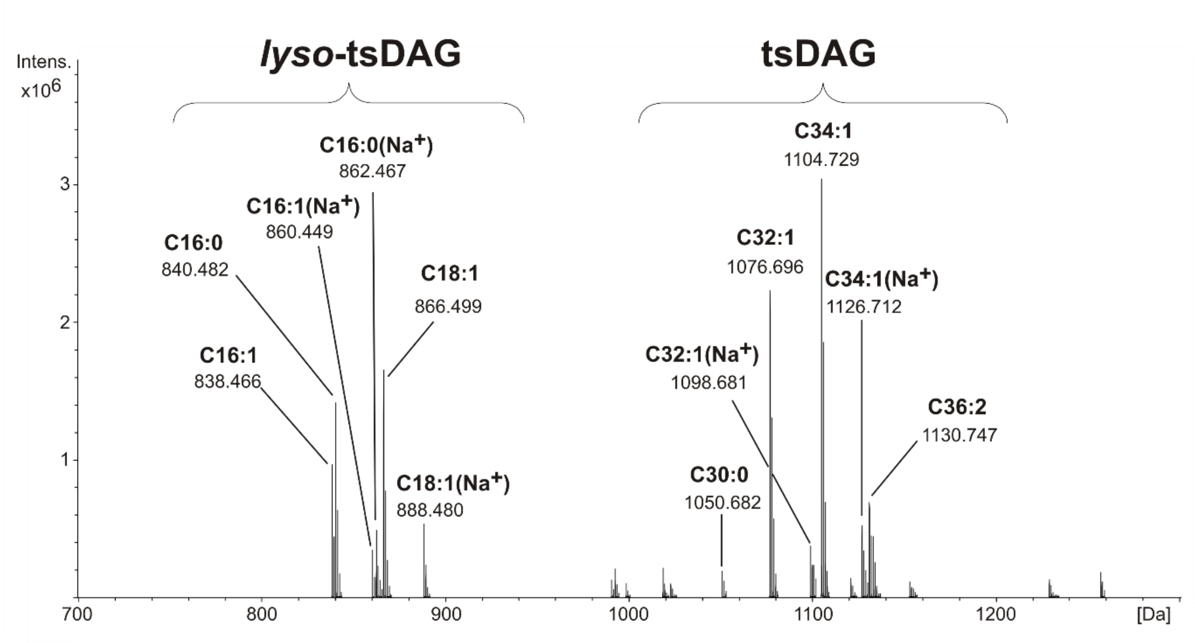

Figure S2. Section of the charge deconvoluted ESI-FT-ICR-MS spectrum (acquired in positive ion mode) of the pool comprising the lipid anchor-containing tsDAG **5** and respective monoacylated variants (*lyso*-tsDAG) obtained by HIC purification (fractions 27-34; Fig. S1B) after 2 d HF treatment of *S. oralis* Uo5Δ*cps* LTA.

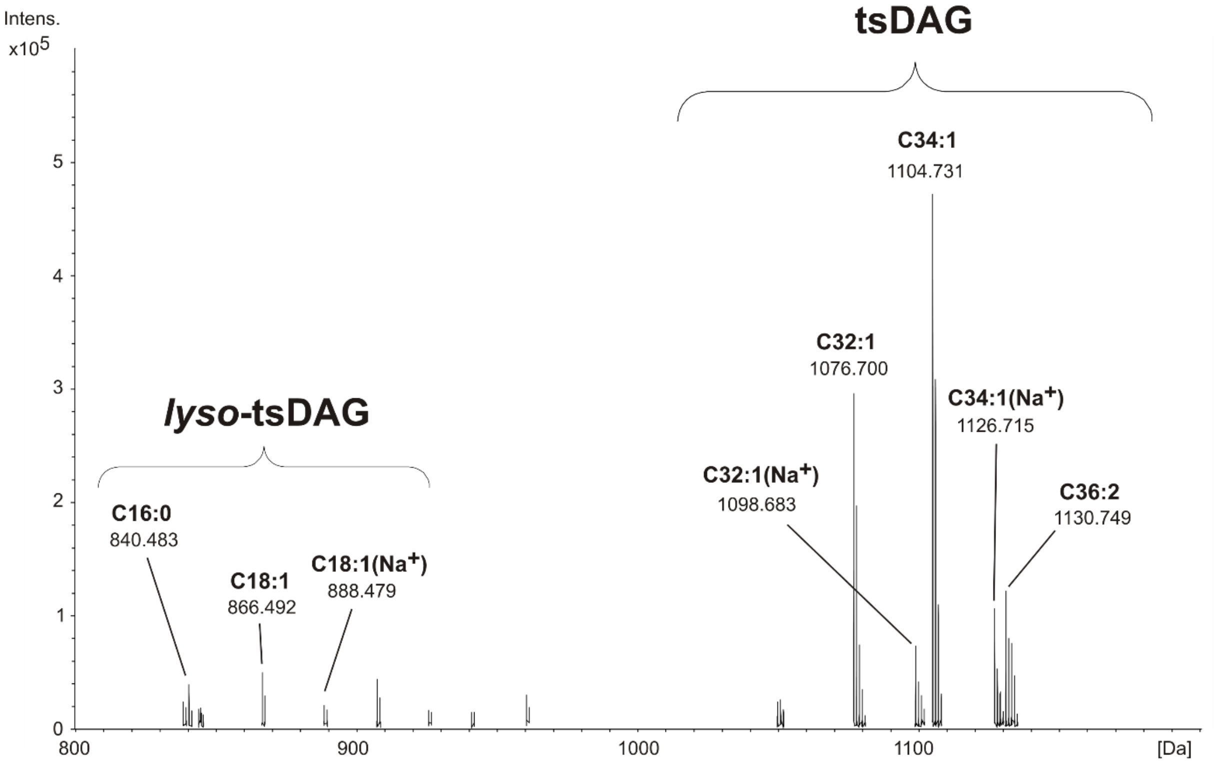

Figure S3. Section of the charge deconvoluted ESI-FT-ICR-MS spectrum (acquired in positive ion mode) after 8 h HF treatment of *S. oralis* Uo5Δ*cps* LTA.

Table S1. ^{1}H (700.4 MHz) and ^{13}C NMR (176.1 MHz) chemical shift data (δ, ppm) [*J*, Hz] for **5** isolated from *S. oralis* Uo5Δ*cps* LTA after 2 d HF treatment (fractions 27-34; Fig. S1B). Values for fatty acid residues and the additionally present *lyso*-compounds (appr. 25%) are not listed. *non-resolved multiplet.

Sugar residue	H-1<br>*C-1*	H-2<br>*C-2*	H-3<br>*C-3*	H-4<br>*C-4*	H-5<br>*C-5*	H-6<br>*C-6*	NAc
β-D-Gal*p*-(1→	4.32 [7.7]	3.54 [9.8, 8.0]	3.44 [9.8, 3.3]	3.81-3.77*	3.52-3.47*	3.71-3.66*/ 3.75 [11.6, 7.2]	
	106.4	*72.2*	*74.4*	*70.1*	*76.8*	*62.5*	
→3)-β-AATGal*p*-(1→	4.65 [8.5]	3.92 [10.8, 8.5]	3.88-3.84*	3.29-3.26*	3.83-3.77*	1.32-1.28*	1.98
	103.3	*52.5*	*81.5*	*55.1*	*70.7*	*16.9*	*23.1*<br>*174.5*
→3)-α-D-Glc*p*(1→	4.79 [3.6]	3.52-3.48*	3.68-3.64*	3.39-3.33*	3.62-3.57*	3.70-3.65*/ 3.82-3.77*	
	100.4	*72.4*	*84.4*	*69.7*	*73.3*	*62.2*	
→3)-Gro(acyl)$_2$	4.21 [12.6, 6.7]/ 4.47 [12.1, 3.8]	5.28-5.24*	3.68-3.62*/ 3.90-3.86*				
	63.5	*71.3*	*66.9*				

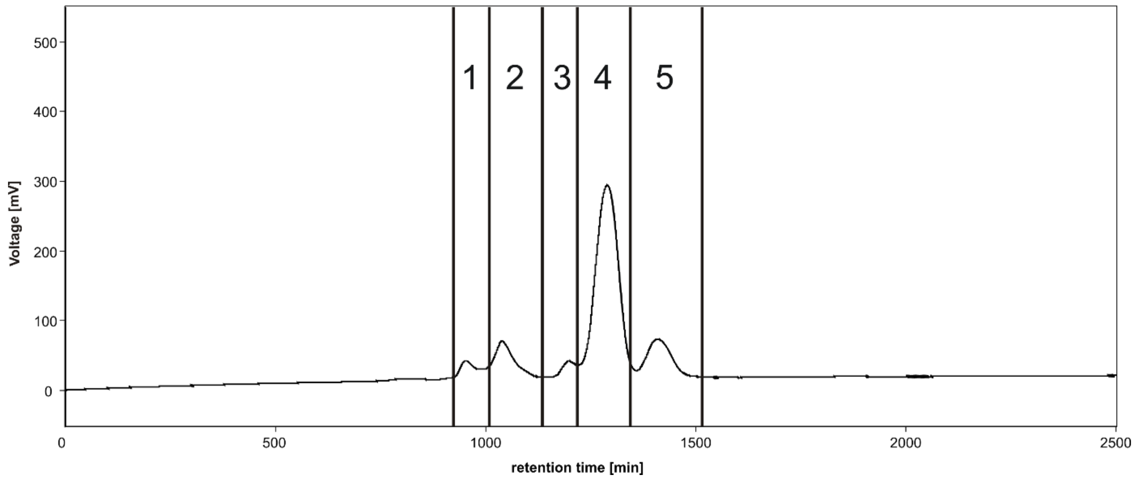

Figure S4. Representative chromatogram of a gel permeation chromatography (GPC; Bio-Gel P-10 column, 150 mM ammonium acetate (pH 4.7)) of the monomerized polysaccharide units containing pool after HIC purification (fractions 7-11; Fig. S1B) of 2 d HF treated LTA. Pool 1 contained **6a** and **6b**, pool 2 **7a** and **7b**, respectively.

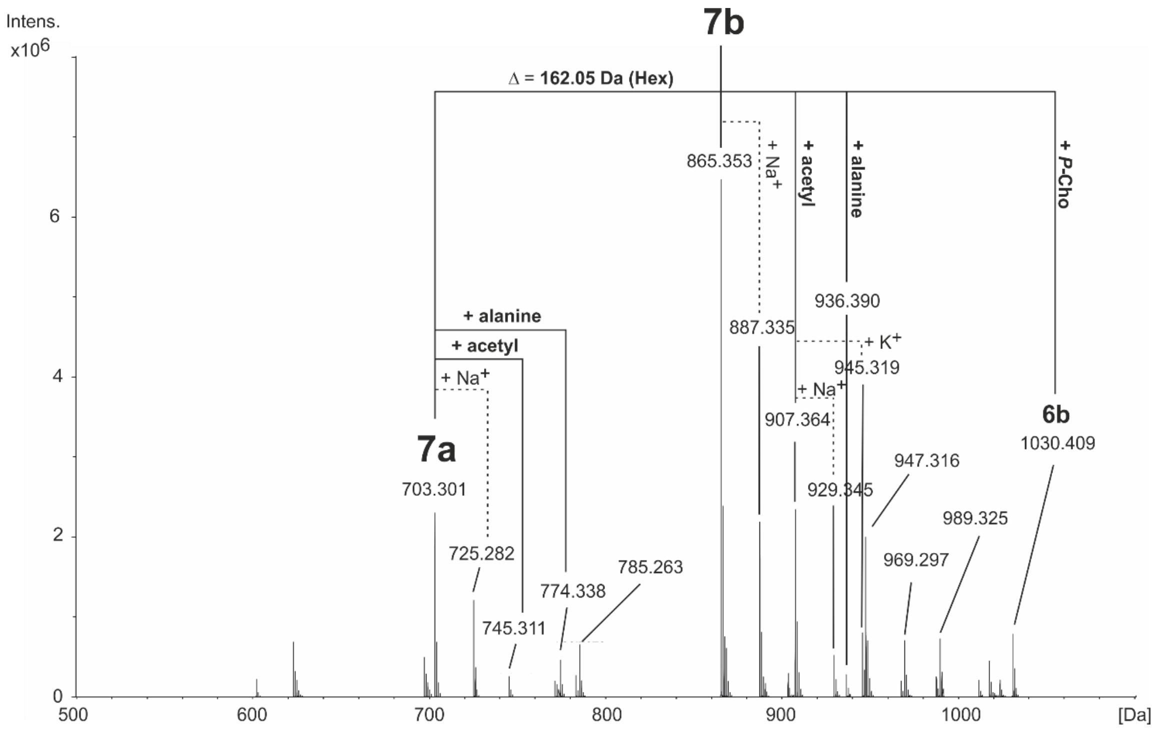

Figure S5. Section of the charge deconvoluted ESI-FT-ICR-MS spectra (acquired in positive ion mode) of **7a,b** obtained after 2 d HF treatment of LTA of *S. oralis* Uo5Δ*cps* and subsequent purification by HIC and GPC (P-10 pool 2 in Fig. S4). Besides the major molecule **7b** with a MW_{found} of 865.353 Da (MW_{calc}: 865.347 Da), a second compound with one hexose less (**7a**; MW_{found}: 703.301 Da; MW_{calc}: 703.298 Da) was observed. For both molecules, variants with a bound alanine or acetyl residue (Table S2) have been identified with very small signal intensity as well.

Table S2. Summary of selected calculated and observed molecular masses [Da] for the isolated monoPS units of *S. oralis* LTA after 2 d HF treatment (**6a,b**: P-10 pool 1 of Fig. S4, MS spectrum in Fig. 2; **7a,b**: P-10 pool 2 of Fig. S4, MS spectrum in Fig. S5). Structures are shown in Fig. 1.

Compound	Calculated	Observed
**6b**	**1030.403**	**1030.408**
- H^+ + Na^+	1052.385	1052.390
+ *acetyl*	1072.413	1072.421
+ *acetyl* - H^+ + Na^+	1094.395	1094.401
+ *alanine*	1101.440	1101.445
**6a**	**868.353**	**868.356**
- H^+ + Na^+	890.335	890.337
+ *acetyl*	910.364	910.367
+ *alanine*	939.390	939.393
**7b**	**865.347**	**865.353**
- H^+ + Na^+	887.329	887.335
+ *acetyl*	907.358	907.364
+ *acetyl* - H^+ + Na^+	929.340	929.345
+ *alanine*	936.384	936.390
**7a**	**703.298**	**703.301**
- H^+ + Na^+	725.280	725.282
+ *acetyl*	745.308	745.311
+ *alanine*	774.335	774.338

Table S3. ^{1}H (700.7 MHz) and ^{13}C NMR (176.2 MHz) chemical shift data (δ, ppm) [*J*, Hz] for **6a,b** isolated from *S. oralis* Uo5Δ*cps* LTA after 2 d HF treatment (P-10 pool 1 of Fig. S4). *non-resolved multiplet.

**Residue (assignment, abundance)**	**H-1**<br>***C-1***	**H-2**<br>***C-2***	**H-3**<br>***C-3***	**H-4**<br>***C-4***	**H-5**<br>***C-5***	**H-6**<br>***C-6***	**NAc**
α-D-Gal*p*-(1→<br>(**E**, 0.7)	5.13 [3.3]<br>*96.0*	3.86-3.83*<br>*68.9*	3.96-3.92*<br>*69.8*	4.02-3.99*<br>*69.8*	4.21-4.17*<br>*71.4*	3.74-3.70*<br>*61.5*	
→3)-β-D-Gal*p*-(1→<br>(**C**, 0.7)	4.50 [7.7]<br>*105.2*	3.67-3.62*<br>*69.8*	3.75-3.70*<br>*77.8*	4.16-4.13*<br>*65.4*	3.72-3.68*<br>*74.5*	3.77-3.70*<br>*61.6*	
β-D-Gal*p*-(1→<br>(**C**#, 0.3)	4.44 [7.7]<br>*105.3*	3.54-3.50*<br>*71.2*	3.63-3.59*<br>*73.1*	3.92-3.88*<br>*69.2*	3.69-3.66*<br>*74.5*	3.77-3.70*<br>*61.6*	
→3)-β-AATGal*p*-(1→<br>(**B**, 1.0)	4.68-4.64*<br>*102.7*	4.03-3.98*<br>*51.2*	3.95-3.91*<br>*80.7*	3.40-3.34*<br>*54.3*	3.89-3.84*<br>*69.9*	1.28 [5.9]<br>*16.5*	2.06<br>*22.9*<br>*175.6*
**B**#			3.96-3.92*<br>*80.1*				
→4)-β-D-3-*O*-*P*-Cho-Gal*p*NAc (1→<br>(**D**, 1.0)	4.70-4.67*<br>*102.3*	3.97-3.93*<br>*52.6-52.5**	4.24-4.21*<br>*74.6-74.4*	4.23-4.19*<br>*75.9-75.7*	3.66-3.62*<br>*75.3*	3.82-3.78*<br>*61.6*	2.02<br>*23.0*<br>*175.0*
Cho-*P*-(*O*→	4.33-4.27*<br>4.24-4.18*<br>*60.2 [4.9]*	3.69-3.65*<br>*66.6-66.5**	3.22<br>54.6				
→2-ribitol<br>(**Rib-ol**, 1.0)	3.84-3.80*<br>3.70-3.66*<br>*61.0*	3.98-3.94*<br>*81.8*	3.88-3.85*<br>*72.4*	3.76-3.73*<br>*72.1*	3.81-3.77*<br>3.63-3.59*<br>*63.4*		

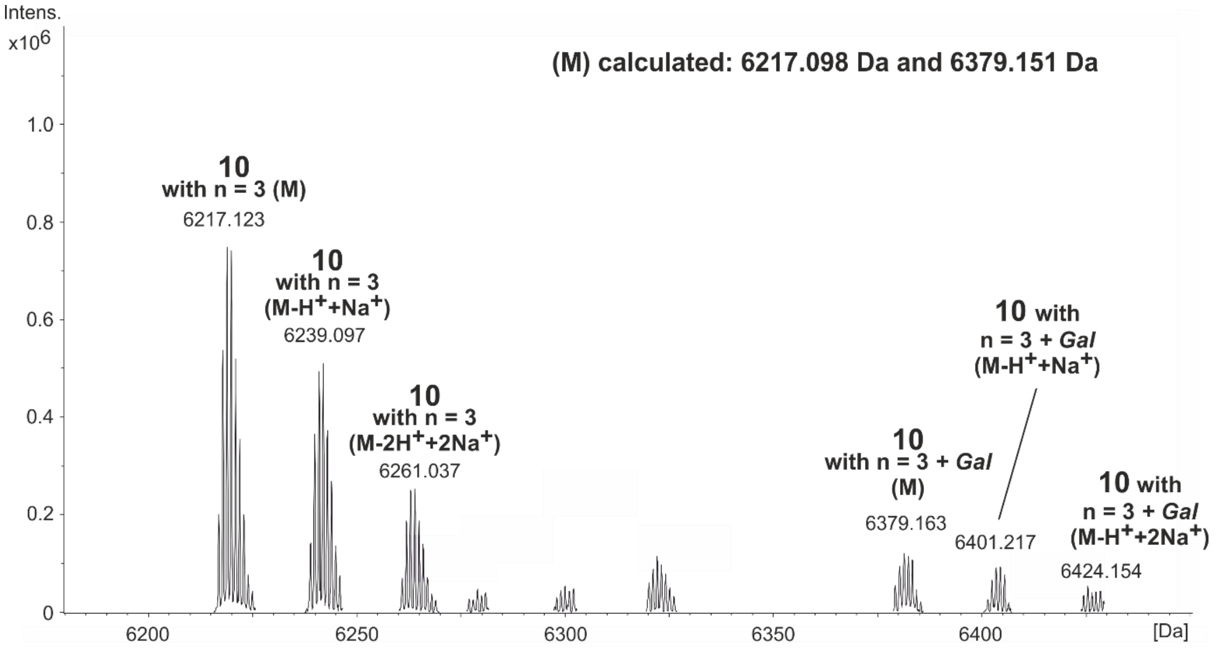

Figure S6. Magnification of the marked section in Fig. 4 (dotted square) of the ESI-FT-ICR-MS spectra of **10** (for structure see Fig. 5A) indicating the presence of a small population of LTA molecules with just one RU without an α-Gal moiety.

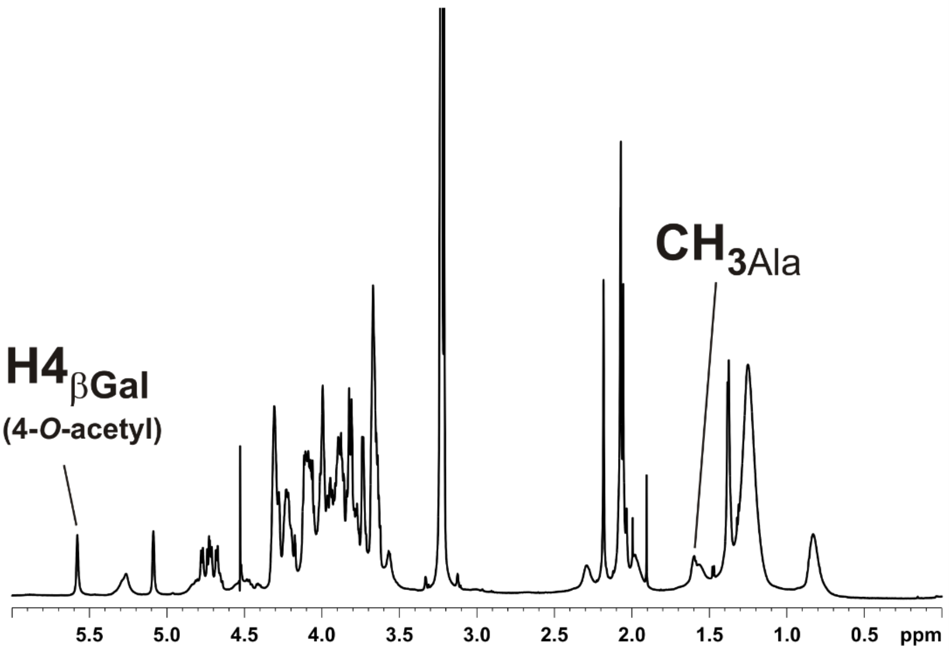

Figure S7. Section (δ_H 6.0-0.0) of the ^{1}H NMR of *S. oralis* Uo5Δ*cps* LTA measured in D_2O at 325 K.

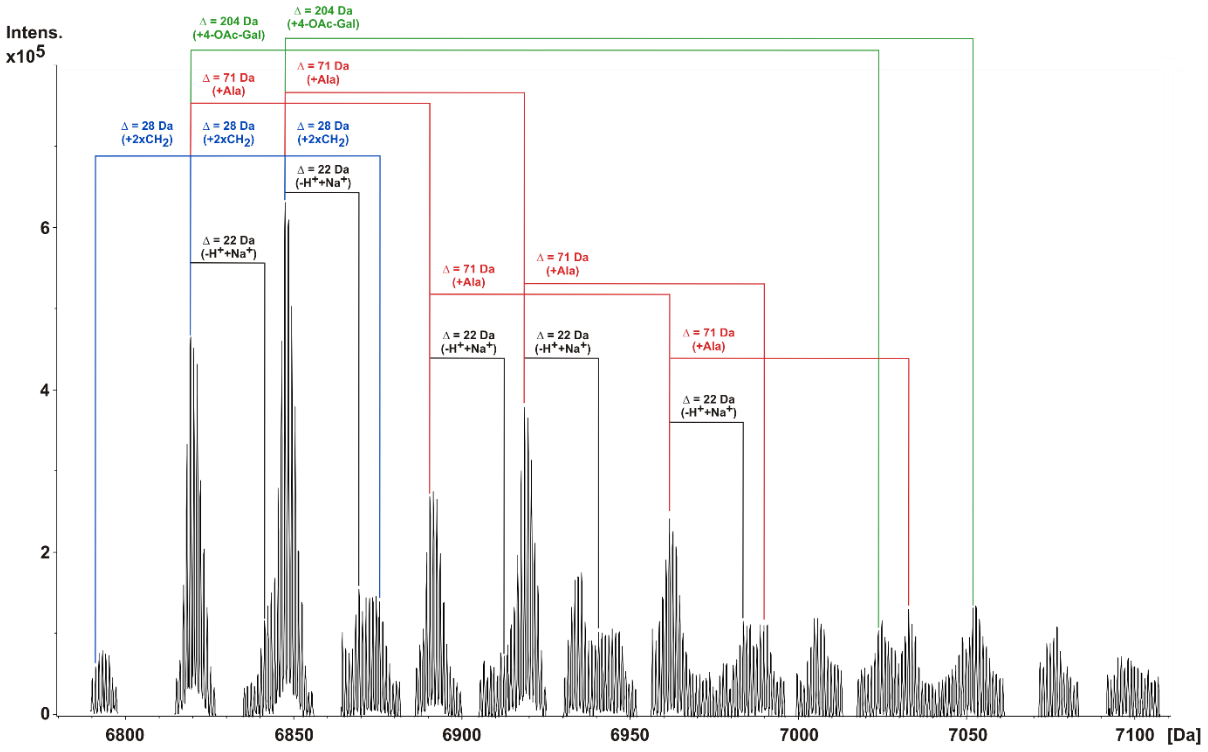

Figure S8. Magnified section of the MS spectra of natural LTA of *S. oralis* Uo5Δ*cps* shown in Fig. 6. The isolated LTA fraction contained molecules with different fatty acid compositions; $(CH_2)_2$-unit differences for fully saturated species are indicated (Δ = 28 Da); molecular species containing one to three alanine substituents (Δ = 71 Da) or one additional 4-OAc-galacose moiety were detected (Δ = 204 Da). Assigned molecular species are listed in Table S4.

Table S4. Assigned molecular species of *S. oralis* Uo5Δ*cps* LTA considering different fatty acid combinations, different numbers of RUs, and potential additional substituents.

LTA species: Gro + Glc +			Monoisotopic mass [Da]		
**Fatty acids**	**RU 1***	**RU 2****	**Calculated** *(sodium adduct)*	**Observed** *(sodium adduct)*	**Mass accuricy [ppm]**
	**2**	**1**			
**32:1**			4218.694	4218.710	3.8
**32:0**			4220.710	4220.723	3.1
**34:2**			4244.710	4244.718	1.9
**34:1**			4246.725	4246.755	7.1
**34:0**			4248.741	4248.757	3.8
	**2**	**2**			
**32:1**			5518.118	5518.117	-0.2
**32:0**			5520.134	5520.115	-3.4
**34:2**			5544.134	5544.149	2.7
**34:1**			5546.150	5546.146	-0.7
**34:0**			5548.165	5548.143	-4.0
	**2**	**3**			
**30:0**			6791.527 (*6813.509*)	6791.540 (*n.d.*)	1.9
**32:2**			6815.527 (*6837.509*)	6815.566 (*6837.625*)	5.7 (*17.0*)
**32:1**			6817.543 (*6839.525*)	6817.569 (*6839.579*)	3.8 (*7.9*)
**32:0**			6819.558 (*6841.541*)	6819.546 (*6841.597*)	-1.8 (*8.2*)
+ 1 Ala			6890.596 (*6912.578*)	6890.661 (*6912.680*)	9.4 (*14.8*)
+ 2 Ala			6961.633 (*6983.615*)	6961.634 (*6983.604*)	0.1 (*-1.6*)
+ 3 Ala			7032.670 (*7054.652*)	7032.577 (*7054.598*)	-13.2 (*-7.7*)
+ 4-OAc-Gal			7023.618 (70*45.601*)	7023.578 (70*45.494*)	-5.7 (*-15.2*)
**34:2**			6843.558 (*6865.541*)	6843.615 (*6865.614*)	8.3 (*10.6*)
**34:1**			6845.574 (*6867.556*)	6845.600 (*6867.622*)	3.8 (*9.6*)
**34:0**			6847.590 (*6869.572*)	6847.649 (*6869.631*)	8.6 (*8.6*)
+ 1 Ala			6918.627 (*6940.609*)	6918.684 (*6940.636*)	8.2 (*3.9*)
+ 2 Ala			6989.664 (*7011.646*)	6989.614 (*n.d.*)	-7.2
+ 4-OAc-Gal			7051.650 (*7073.632*)	7051.600 (*7073.535*)	-7.1 (*-13.7*)
**36:2**			6871.590 (*6893.572*)	6871.640 (*6893.669*)	7.3 (*14.1*)
**36:1**			6873.605 (*6895.588*)	6873.648 (*6895.631*)	6.3 (*6.3*)
**36:0**			6875.621 (*6897.603*)	6875.660 (*6897.658*)	5.8 (*8.0*)
	**2**	**4**			
**32:1**			8116.967	8116.895	-8.9
**32:0**			8118.983	8118.901	-10.1
**34:2**			8142.983	8142.914	-8.5
**34:1**			8144.999	8144.894	-12.9
**34:0**			8147.014	8146.952	-7.6

*RU 1 = 3,6-di-*O*-*P*-Cho-β-D-Gal*p*NAc-(1→2)-Rib-ol-1-*P*-(*O*→6)-β-D-Gal*p*-(1→3)-AATGal*p* (**8**)
RU 2 = 3,6-di-*O*-*P*-Cho-β-D-Gal*p*NAc-(1→2)-Rib-ol-1-*P*-(*O*→6)-(3-α-D-Gal*p*,4-OAc)-β-D-Gal*p*-(1→3)-AATGal*p* (9b**).

Table S5. Oligonucleotides used in this study.

Name	Sequence (5'-3')
U05_cps_pcr1_f	CCGTCAGGCTATCAACTTTGGTATTGATCG
U05_cps_pcr1_r	CCTCTGGAATAGGCGTCGACGCGAATAATCCCCTATACACGTTAATAG
U05_cps_pcr2_f	TAACGTGTATAGGGGATTATTCGCGTCGACGCCTATTCCAGAGG
U05_cps_pcr2_r	GATTAATGAGTAACTTCAAATTCCTTCAGAAAAGATTAGATGTC
U05_cps_pcr3_f	GACATCTAATCTTTTCTGAAGGAATTTGAAGTTACTCATTAATCAG
U05_cps_pcr3_r	GTCGATGACCTACCTGATCCGCAAACAGAG
